# Combined and synergistic effects of heat and fine particulate matter on hospitalization among patients with Alzheimer’s disease and related dementias

**DOI:** 10.1097/EE9.0000000000000440

**Published:** 2025-11-21

**Authors:** Federica Spoto, Antonella Zanobetti, Scott W. Delaney, Thomas M. Gill, Michelle L. Bell, Francesca Dominici, Danielle Braun, Daniel Mork

**Affiliations:** a Department of Biostatistics, Harvard T.H. Chan School of Public Health, Boston, Massachusetts; b Department of Environmental Health, Harvard T.H. Chan School of Public Health, Boston, Massachusetts; c Department of Internal Medicine, Yale School of Medicine, New Haven, Connecticut; d School of the Environment, Yale University, New Haven, Connecticut; e Department of Data Science, Dana Farber Cancer Institute, Boston, Massachusetts

**Keywords:** Air pollution, Particulate matter, Short-term exposure, Temperature, Risk assessment, Alzheimer’s disease and related dementias

## Abstract

**Background:**

Patients with Alzheimer’s disease and related dementias (ADRD) are vulnerable to environmental stressors such as extreme heat and air pollution, yet their combined health effects remain poorly understood.

**Methods:**

We assessed the joint impact of extreme heat and fine particulate matter exposure (PM_2.5_) on the risk of all-cause hospitalization among an ADRD cohort of Medicare beneficiaries aged ≥65 years. Using a time-stratified case-crossover design, we analyzed data from beneficiaries with prior ADRD-related hospitalizations across the contiguous US in 2000–2016. Daily heat index and PM_2.5_ concentrations were linked to residential ZIP codes, and conditional logistic regression models were applied to estimate same-day associations during the warm season (May–September), including interaction terms to explore potential synergistic effects.

**Results:**

We found a linear association between heat and hospitalization, with an odds ratio (OR) of 1.017 (95% confidence interval [CI] = 1.004, 1.031) on extreme heat days (99th percentile) versus median. The PM_2.5_-hospitalization relationship was nonlinear, with stronger effects at lower concentrations (10 vs. 5 µg/m^3^ OR = 1.010; 95% CI = 1.005, 1.015). When accounting for changes in PM_2.5_, the OR on extreme heat days versus the median was 1.016 (95% CI = 1.001, 1.032).

**Conclusion:**

These findings underscore the need to consider both environmental stressors when assessing health risks in ADRD populations.

**What this study adds**

This study is the first to evaluate both the independent and combined short-term effects of extreme heat and PM_2.5_ on hospitalization risk among individuals with Alzheimer’s disease and related dementias in a nationwide US cohort. Our findings provide new evidence of synergistic effects, emphasizing the need for integrated public health interventions targeting climate and air pollution risks in this vulnerable population.

## Introduction

Alzheimer’s disease (AD) currently affects 6.9 million people in the US, is the fifth-leading cause of death for adults age 65 and over, and in 2024 alone, the costs for care were estimated to exceed $360 billion.^1^ Alongside a rapidly aging population, the incidence of AD is expected to grow to over 13.8 million by the year 2060.^1–5^ Individuals with AD and related dementias (ADRD) are also highly vulnerable due to multiple comorbidities including heart disease, diabetes, and kidney disease.^6–11^ The complex nature of ADRD makes individuals with ADRD potentially more susceptible to health impacts due to a range of environmental exposures, such as extreme heat and air pollution. However, environmental risks are often addressed through isolated policies, and exposures are typically studied independently, resulting in an incomplete picture of the risks associated with their combined effects. A clear understanding of the independent or synergistic effects of such exposures can lead to more informed policy and health decisions to best mitigate risks among the ADRD population.

In this work, we focus on two environmental risk factors: extreme heat exposure and fine particulate matter air pollution (PM_2.5_), that is, airbourne particles no larger than 2.5 µm in diameter. Extreme heat can cause dehydration and heat stroke, which can lead to respiratory distress and hospitalizations.^12^ PM_2.5_, when inhaled, can pass from the lungs to the bloodstream, promoting reactive oxygen species and oxidative stress, which may lead to adverse health effects, including cardiovascular and respiratory diseases.^13–16^ Persons with ADRD might be more vulnerable to the effects of heat and air pollution because their symptoms and limitations hinder their ability to stay cool or access cleaner air.^11,17–19^ For example, individuals with ADRD may struggle to stay hydrated or cool due to difficulties with thermoregulation or cognitive impairments,^20^ increasing their risk of heat-related illnesses like heat exhaustion or heatstroke; additionally, cognitive and physical limitations can prevent them from using air purifiers, exacerbating respiratory issues that may arise from poor air quality.

Several studies have reported statistically significant evidence of an association between short-term exposure to heat and higher rates of ADRD-related hospitalization and a higher mortality risk among individuals with ADRD.^17,21–26^ Fewer studies have explored the impact of short-term exposure to PM_2.5_ in individuals with ADRD. Franco et al.^27^ and Zhang et al.^28^ found a positive significant association between short-term exposure to PM_2.5_ and increased ADRD-related hospital admissions in New York and Lisbon, both controlling for temperature, while Zanobetti et al.^29^ found a positive significant association between short-term exposure to PM_2.5_ and all-cause mortality in subjects with previous hospital admissions for ADRD.

The relation between heat and PM_2.5_ is complex and varies based on several conditions, such as seasonality, regional differences, and wind speed.^30–32^ A positive correlation has been found between temperature and PM_2.5_, especially during summer, which may reflect the fact that higher temperatures can enhance photochemical reactions increasing PM_2.5_ concentrations.^33–35^ Also, higher PM_2.5_ can cooccur with high temperature due to higher likelihood for stagnant air masses and increased risk of wildfires.^30^ While research typically adjusts for temperature in air pollution-health studies,^27–29^ and vice versa,^21,23,25^ the joint effects and potential interactions between these variables are not well understood. Existing research on the synergistic effects of PM_2.5_ and heat index has primarily focused on mortality,^36–38^ preterm birth,^39,40^ and cause-specific hospitalizations, such as respiratory, cardiac, and stroke admissions.^41^ For instance, Rahman et al.^36^ found a higher risk of all-cause mortality for days when both exposures were at extreme levels compared with only one exposure at an extreme level, through a case-crossover study in California that analyzed the interaction between the two exposures. However, to the best of our knowledge, no research has explored the potential interactions between PM_2.5_ and extreme heat in an ADRD cohort. Studies by Culqui et al.^42^ and Linares et al.^43^ assessed the independent effects of heat and PM_2.5_ on hospital admissions for AD and dementia in Madrid, considering additional environmental factors, but omitted potential interactions. Our study focuses on analyzing the possible interaction effect between PM_2.5_ and heat. We do not carry out a mediation analysis, but, through an additional exploratory analysis, we account for the relationship between the exposures. Investigating these interactions is vital for identifying any synergistic effects or verifying their independence, especially for vulnerable populations like those with ADRD.

We conducted a time-stratified case-crossover analysis among Medicare enrollees with a previous hospitalization with an ADRD diagnosis code. Medicare is a US federal program that provides health insurance to individuals aged 65 and older. We linked daily heat index and PM_2.5_ data to study the synergistic effects of extreme heat and PM_2.5_ exposure on all-cause hospitalization during the warm season (from May to September). Our study analyzed the same-day exposure, allowing for linear and nonlinear exposure-response relationships. We began by assessing the effects of each exposure separately, next evaluating them in a joint modeling framework, and ultimately considering their interaction. The study was carried out in the contiguous US during the 17-year period from 2000 to 2016.

## Methods

### Study population

We obtained Medicare Part A claims data from the Centers for Medicare and Medicaid Services and included all Medicare fee-for-service enrollees aged 65 and older from 1 January 2000 to 31 December 2016. These data include information at the individual level on age, sex, self-reported race and ethnicity, Medicaid eligibility (Medicaid is a joint federal and state program in the US that offers health coverage to low-income individuals), and annually updated residential ZIP code for each enrollee. Each enrollee had a unique identifier for tracking.

We aim to evaluate incident all-cause hospitalizations following cohort entry in individuals with ADRD. We define a qualifying hospitalization as the first hospitalization with at least one ADRD-related diagnosis code in the first ten billing codes, based on the refined definition by Moura et el.^44^ (see Table S1 in Supplementary Material; https://links.lww.com/EE/A386). We specify our ADRD cohort as the cohort of enrollees who have a qualifying hospitalization during the study period across the contiguous US. Among our ADRD cohort, we define our outcome to be the first all-cause hospitalization during the warm season (May to September) after entering the cohort, allowing us to examine heat exposure effects. Hence, the qualifying hospitalization is solely used to identify the cohort of interest for the study, while hospitalizations within this cohort define the ’cases’ in our analysis. The case day is defined as the admission day of the outcome hospitalization. We included only enrollees whose outcome hospitalization occurred at least 30 days after their qualifying hospitalization and without another admission within 30 days from the outcome hospitalization discharge. The target population is composed of enrollees with mild disease severity, and this washout period helps to ensure that our outcome reflects hospitalizations not directly related to the qualifying admission and reduces the potential for confounding by disease severity or instability at cohort entry. We excluded enrollees admitted from skilled nursing facilities for their outcome hospitalization. The rationale for excluding enrollees coming from a skilled nursing facility is to avoid including patients who might be shielded from exposure and thus not be subject to the underlying mechanism of the exposure-hospitalization relationship. Hospitalizations were included if the beneficiary was admitted from a nonhealthcare facility, via clinic referral, or through the emergency room, covering over 91% of cases. Further detail on the inclusion criteria is provided in the Supplementary Materials Section S2; https://links.lww.com/EE/A386.

### Exposure data: PM_2.5_ and heat index

We obtained daily PM_2.5_ exposure levels across the US, predicted at a 1 km2 grid resolution based on an ensemble modeling framework with temporal R^2^ of 0.85.^45^ The distribution of PM_2.5_ is highly right-skewed, and for this reason, when presenting the results, we do not make inference beyond the 99th percentile (36.18 µg/m^3^) as there is high uncertainty at the higher levels. When describing the results, we focused only on values up to 15 µg/m^3^, which is the World Health Organization’s recommended daily (24-hour average) limit for PM_2.5_.

**Figure 1.**
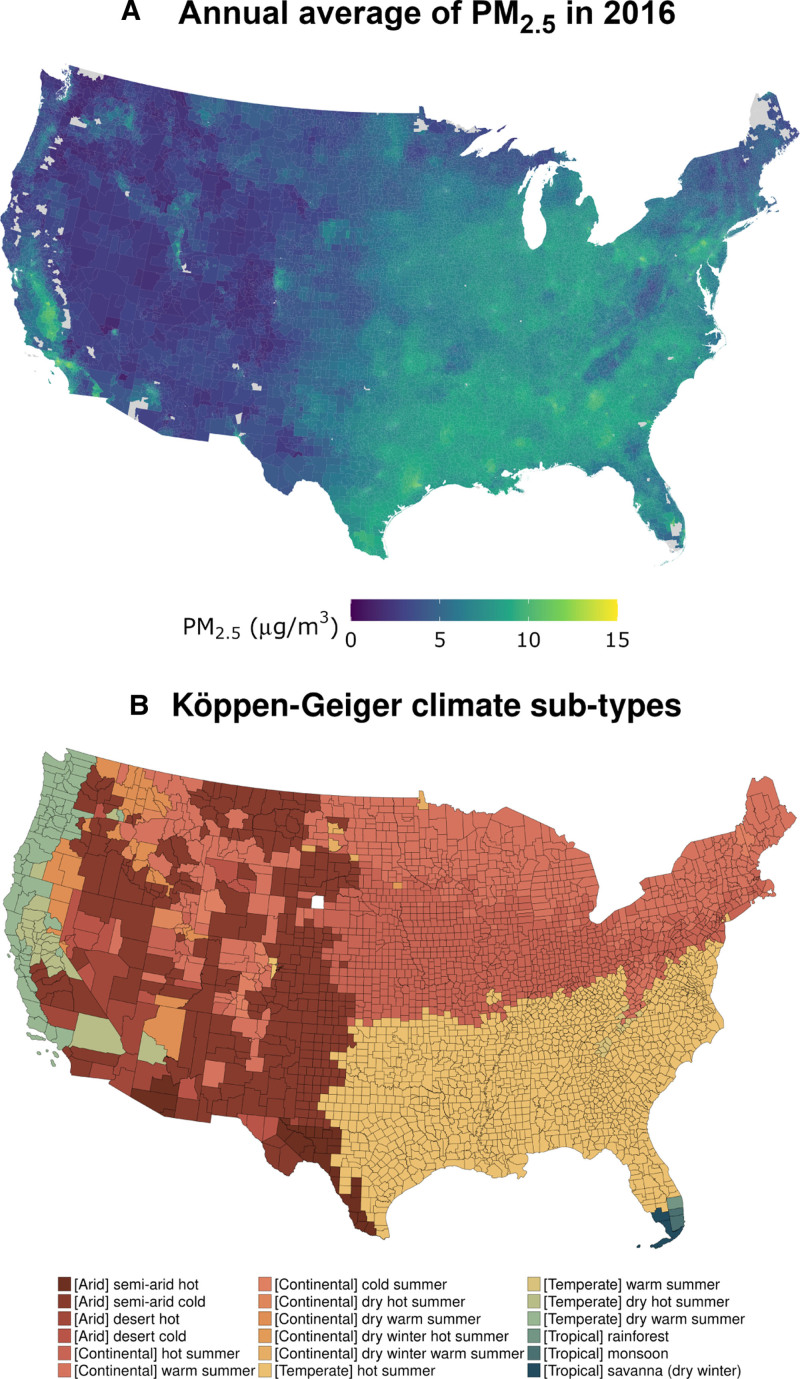
Köppen–Geiger climate subtypes and air pollution maps. Panel (A) shows the annual average PM_2.5_ at the ZIP code level in 2016. Panel (B) visualizes the 18 Köppen–Geiger climate subtypes represented in the contiguous US by county.

Daily ZIP code PM_2.5_ levels were obtained by averaging all grid centroids within ZIP code boundaries. Figure 1A shows the annual average level of PM_2.5_ at the ZIP code level for the year 2016. Daily maximum temperature and minimum relative humidity at 4 km2 resolution were obtained from gridMET and aggregated at the ZIP code level by averaging all grid centroids within ZIP code boundaries to match the resolution of Medicare data.^46^ We calculated the daily maximum heat index, which combines temperature and humidity to indicate perceived heat, following the definition given by the US National Weather Service. We specifically used the R package weathermetrics, whose equations are from the source code for the US National Weather Service’s online heat index calculator.^47,48^ Heat index reflects perceived rather than absolute temperature, accounting for potential acclimatization based on local climate.

To account for differing perceptions of temperature based on climate, enrollees were assigned Köppen–Geiger climate subtypes based on their residential county. The contiguous US has four climate types (Arid, Continental, Temperate, Tropical) and 18 subtypes, which classify regions by temperature and precipitation patterns, as shown in Figure 1B. We calculated heat index percentiles specific to each climate subtype’s warm-season heat index distribution, ranging from 0% to 100%. The percentiles were calculated across both time and space; specifically, they were derived for each Köppen–Geiger climate subtype using all warm season measurements over the study period (2000–2016), incorporating both case and control days. By calculating percentiles within climate subtypes, we aim to partially account for differences in local temperature distributions and population acclimatization.

We refer to days at or above the 99th percentile as “extreme heat days” throughout this manuscript.

### Statistical analysis

We conducted a time-stratified case-crossover analysis to examine the short-term main association with and without interaction of heat and fine particulate matter on all-cause hospitalizations among our ADRD cohort.^49^ This self-matching study design compares exposures on hospitalization (case) days to exposures on matched control days, effectively controlling for all time-invariant individual and area-level factors, like demographic characteristics. Control days were defined as the same day of the week within the same month and year, employing a mixed matching of bidirectional and unidirectional controls. For instance, if hospitalization occurred on a Monday in July 2014, other Mondays in July 2014 served as control days. Defining control days within the same month and year helps isolate the exposure effect by controlling for temporal trends in PM_2.5_ across years.

We estimated the association between same-day exposures and hospitalization using conditional logistic regression. Initially, single-exposure analyses independently assessed the relationship between each same-day exposure and all-cause hospitalization, considering both linear and nonlinear associations. Single-exposure models were evaluated to replicate and confirm previous results within our cohort, and to provide a baseline for following analyses. We then examined their joint association with all-cause hospitalizations by first considering the two exposures independently and then including an interaction term to determine if combined exposures exacerbate health impacts. Similar to the single-exposure analyses, we ran joint-exposure analyses considering both linear and nonlinear associations with the hospitalization rate. Our primary aim was to describe association patterns and evaluate whether an interaction effect exists between heat effects and PM_2.5_.

Because previous literature found a positive correlation between heat and PM_2.5_,^33–35^ we also investigated and included this relationship. Specifically, we first estimated the association between the heat index and PM_2.5_, independently from the hospitalizations. Subsequently, we utilized these estimates to predict PM_2.5_ levels based on the heat index, which were then incorporated into our assessment of their combined impact on hospital admissions. While not a formal mediation analysis, which is beyond the scope of this study, this additional analysis accounts for the relationship between the two exposures.

For the single exposure models and multiple exposures model without interaction, we modeled the nonlinear effect of heat index using B-splines (BS) with 4 degrees of freedom (df) and the nonlinear effect of PM_2.5_ using BS with 4 df. In the joint-exposure analysis with interaction, we constructed a crossbasis based on BS with 3 df for each exposure to model the heat-PM_2.5_ interaction.^50^ The choice of the degrees of freedom has been carried out by minimizing the Akaike information criterion.

Finally, we conducted an analysis to account for the interdependence between the two environmental exposures. Initially, we employed a B-spline regression model with 4 degrees of freedom, based on the Akaike information criterion, to estimate the dependency of PM_2.5_ levels on heat index values. From this model, we predicted PM_2.5_ concentrations at specific heat index values of interest, such as when the heat index reaches the 99th percentile. By integrating these predicted PM_2.5_ levels with the previously described nonlinear interaction model, we calculated a contrast to evaluate the expected change in PM_2.5_ associated with a specific change in the heat index. Additional details on this analysis can be found in the Supplementary Materials Section S3; https://links.lww.com/EE/A386.

Results are presented as odds ratios (OR) for all-cause hospital admissions, with reference levels of 5 µg/m^3^ for PM_2.5_ (11.5% percentile) and median heat index. Confidence intervals (CIs) for single- and multiple-exposure models were calculated directly from model outputs. For the analysis incorporating expected PM_2.5_ changes based on heat index levels, we used a Monte Carlo procedure with 5,000 iterations to account for uncertainty from two models. Additionally, we conducted a sensitivity analysis, detailed in the Supplementary Materials Section S4; https://links.lww.com/EE/A386, where we replicated the analyses using a 3-day moving average of heat index and PM_2.5_ as exposure variables. Lastly, we conducted a sensitivity analysis, detailed in the Supplementary Materials Section S4; https://links.lww.com/EE/A386 by rerunning the linear interaction model in two temporal subcohorts, defined by the year of hospitalization. The first subcohort included individuals hospitalized between 2000 and 2007, and the second covered 2008 to 2016. This approach allowed us to assess whether the declining trend in PM_2.5_ levels over time may have influenced or confounded the associations observed in our main analysis.

## Results

During our study period across the contiguous US, over 8 million Medicare enrollees had a qualifying hospitalization with at least one ADRD-related billing code. Among those 8 million, 3.59 million had an all-cause hospitalization at least 30 days after their qualifying hospitalization, of which 1,457,109 occurred during the warm season. After applying our exclusion criteria (detailed in the Supplementary Materials Section S2; https://links.lww.com/EE/A386), our study focused on 713,007 Medicare enrollees across 30,306 ZIP codes. The cohort predominantly consisted of females (65.6%), White individuals (82.6%), and those ineligible for Medicaid (69.9%). The average age of enrollees on the case day was 83 years (SD = 7.2). Table 1 details the cohort characteristics. The average heat index over the study period was 28.86 °C (interquartile range = 24.87 to 33.48 °C), while the average PM_2.5_ was 11.82 µg/m^3^ (interquartile range = 6.80 to 15.00 µg/m^3^).

**Table 1.** Characteristics of Medicare enrollees aged 65 years and older who had an all-cause hospitalization during the warm season after a qualifying hospitalization to enter the ADRD cohort

Enrollees’ characteristics	N (%)
# Enrollees with all-cause hospitalizations in our ADRD cohort	713,007 (100%)
Sex	
Female	467,819 (65.6%)
Male	245,188 (34.4%)
Age	
65–69	35,053 (4.9%)
70–74	61,941 (8.7%)
75–79	115,631 (16.2%)
80–84	176,912 (24.8%)
85–89	186,310 (26.1%)
90–94	108,623 (15.2%)
95 or above	28,537 (4.0%)
Race and ethnicity	
American Indian and Alaska Native	2,388 (0.3%)
Asian and Pacific Islander	7,046 (1.0%)
Black	92,254 (12.9%)
Hispanic	16,089 (2.3%)
Non-Hispanic White	589,217 (82.6%)
Other and unknown	6,013 (0.9%)
Medicaid eligibility	
Ineligible	498,298 (69.9%)
Eligible	214,709 (30.1%)
Köppen–Geiger climate types	
Arid	41,354(5.8%)
Continental	297,324 (41.7%)
Temperate	358,643 (50.3%)
Tropical	15,686 (2.2%)
Air pollution and meteorological variables	Median (SD)
PM_2.5_, µg/m^3^	
Case days	10.2 (7.1)
Control days	10.2 (7.1)
Heat index, °C	
Case days	29.2 (6.3)
Control days	29.1 (6.3)

### Single exposure analysis

In the single-exposure analyses, heat index was significantly associated with increased all-cause hospitalization in our ADRD cohort. For PM_2.5_, a significant association was observed only in the nonlinear analysis, as detailed in Table 2. Assessing the linear relationship, we estimated an OR of 1.014 (95% CI = 1.008, 1.021) for hospitalization on extreme heat days (99th percentile, i.e., a 49% percentile increase in heat exposure) versus median temperature days and an OR of 1.002 (95% CI = 1.000, 1.004) for a 5 µg/m^3^ increase in PM_2.5_.

**Table 2.** Odds ratio for all-cause hospitalization

	Model	Heat index	PM_2.5_
Single exposure	Linear	1.014 (1.008, 1.021)	1.002 (1.000, 1.004)
	Nonlinear	1.017 (1.004, 1.031)	1.010 (1.005, 1.015)
Joint-exposure	Linear	1.015 (1.007, 1.022)	1.000 (0.997, 1.002)
	Nonlinear	1.017 (1.003, 1.031)	1.005 (1.000, 1.011)

The table presents the odds ratio for all-cause hospitalization in moving from the median level of heat index to extreme heat days (99th percentile) and for a 10 µg/m^3^ level of PM_2.5_ compared with 5 µg/m^3^ of PM_2.5_. The odds ratios are estimated both using single-exposure models and joint-exposure model without interaction.

**Figure 2.**
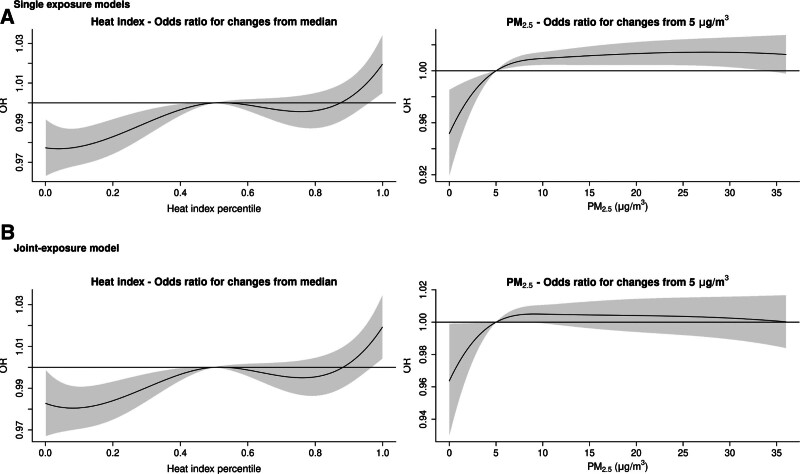
Odds ratio for all-cause hospitalization. The plots present the estimated nonlinear odds ratio for all-cause hospitalization in comparing to the median level of heat index and 5 µg/m^3^ of PM_2.5_. Panel (A) shows the odds ratio obtained from the single-exposure models, panel (B) shows the odds ratio obtained from the joint-exposure without interaction model.

The analysis of nonlinear relationships, shown in Figure 2A and Table 2, indicated an approximately linear trend for heat index percentiles, with an OR of 1.017 (95% CI = 1.004, 1.031) for all-cause hospitalization on extreme heat days (99th percentile) versus median temperature days. The PM_2.5_-all-cause hospitalization relationship had a steeper slope at lower concentrations, flattening above 10 µg/m^3^. The OR for all-cause hospitalization at 10 versus 5 µg/m^3^ was 1.010 (95% CI = 1.005, 1.015); at 15 versus 10 µg/m^3^, was 1.002 (95% CI = 0.998, 1.006). Results for both linear and nonlinear associations are presented in Table 2.

### Joint-exposure analysis

The associations estimated from the joint-exposure model without interaction were similar to those from the single-exposure model for both exposures (Table 2). In the linear analysis, the OR for all-cause hospitalization was 1.015 (95% CI = 1.007, 1.022) for an extreme heat day compared with the median heat index and 1.000 (95% CI = 0.997, 1.002) for a 5 µg/m^3^ increase in PM_2.5_. The nonlinear analysis also showed similar relationships to the single exposure analysis (Figure 2). The estimated OR for all-cause hospitalization on extreme heat days versus median days was 1.017 (95% CI = 1.003, 1.031). For PM_2.5_, the OR for 10 versus 5 µg/m^3^ was 1.005 (95% CI = 1.000, 1.011), and for 15 versus 10 µg/m^3^, it was 1.000 (95% CI = 0.996, 1.004).

**Figure 3.**
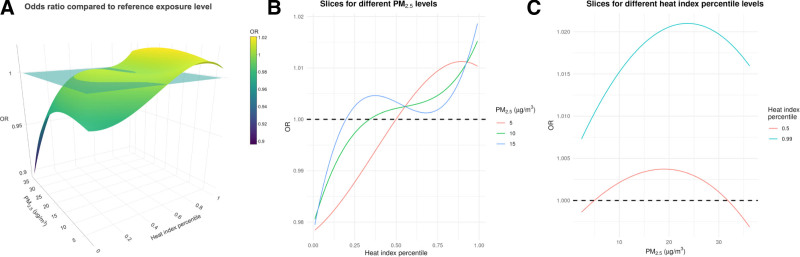
Joint-exposure nonlinear interaction model. The plots present the estimated nonlinear OR for all-cause hospitalizations compared with the median level of heat index and 5 µg/m^3^ of PM_2.5_, accounting for their interaction. Panel (A) shows the OR for the different values of heat index and PM_2.5_ with respect to 5 µg/m^3^ of PM_2.5_ and the median value of heat index, the blue plane shows where the OR is equal to 1. Panels (B) and (C) show slices of the first plot. Specifically, panel (B) shows the OR for exposure levels compared with 5 µg/m^3^ of PM_2.5_ and the median level of heat index, for fixed values of PM_2.5_ equal to 5, 10, and 15 µg/m^3^. Panel (C) shows the OR for exposure levels compared with 5 µg/m^3^ of PM_2.5_ and the median level of heat index, for heat index values fixed at the median, and 99% percentile.

Including an interaction term in the models slightly altered the marginal association between each exposure and odds of all-cause hospitalization. In the linear analysis, the estimated marginal OR for all-cause hospitalization was 1.022 (95% CI = 1.011, 1.033) for extreme heat days compared with the median heat index and 0.997 (95% CI = 0.993, 1.001) for a 5 µg/m^3^ increase in PM_2.5_. The nonlinear analysis also indicated a higher all-cause hospitalization risk with higher exposure levels. Figure 3 illustrates the OR associated with variations in exposure as compared with the reference levels, defined as 5 µg/m^3^ for PM_2.5_ and the median heat index. The first panel presents a comprehensive view of these changes using a 3D plot. The second and third panels provide detailed insights by slicing the data based on specific values of interest for PM_2.5_ and the heat index, respectively. Specifically, panel (B) shows the OR for exposure levels compared with 5 µg/m^3^ of PM_2.5_ and the median level of heat index, for heat index values fixed at the median and 99% percentile. Panel (C) shows the OR for exposure levels compared with 5 µg/m^3^ of PM_2.5_ and the median level of heat index, for fixed values of PM_2.5_ equal to 5, 10, and 15 µg/m^3^. Figure S5 in the Supplementary Materials; https://links.lww.com/EE/A386 presents the 95% CIs.

The interaction provides insight into the impact of a fixed change in one exposure depending on the value of the other. For example, the OR was 1.002 (95% CI = 0.995, 1.010) when PM_2.5_ increased from 5 to 10 µg/m^3^ at the 50th heat index percentile, but rose to 1.005 (95% CI = 0.988, 1.021) when evaluating at the 99th percentile heat index. The OR for extreme heat days compared with the median heat index was 1.010 (95% CI = 0.981, 1.040) at 5 µg/m^3^ PM_2.5_, and increased to 1.013 (95% CI = 0.997, 1.030) when the corresponding PM_2.5_ level equals 10 µg/m^3^. We estimated that the joint increase of both exposures increased the risk of all-cause hospitalization; the OR was 1.015 (95% CI = 0.998, 1.033) for extreme heat and 10 µg/m^3^ PM_2.5_ compared with median heat index and 5 µg/m^3^ PM_2.5_. In Figure 3, moving to higher levels of exposure in both panels, we can see how the interaction affects the OR. These results suggest the presence of a synergistic interaction effect, especially when looking at how the effect of exposure to PM_2.5_ varies across different levels of heat index. The trend for increasing exposure of PM_2.5_ is similar, but the ORs are higher during extreme heat days compared with median ones. This suggests that the effect of PM_2.5_ is exacerbated when individuals are also exposed to higher values of the heat index. Note that the apparent drop in OR at the highest PM_2.5_ levels when the heat index is equal to 0 isn’t showing a real trend, but is an artifact caused by a lack of data at those specific combinations of values, as shown in Figure S7 in Supplementary Materials; https://links.lww.com/EE/A386. In Figure 3, panels B and C present slices of panel A, across the three panels, the reference point is always the same (median heat index and 5 µg/m^3^ for PM_2.5_). Figure S8 in Supplementary Materials; https://links.lww.com/EE/A386 provides additional insight, presenting the exposure-response curve for different reference levels.

**Figure 4.**
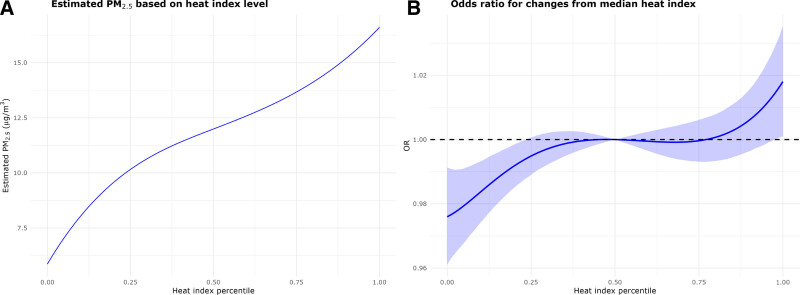
Association between PM_2.5_ and heat index and association with all-cause hospitalizations. Panel (A) shows the association between PM_2.5_ and heat index presenting the estimated values of PM_2.5_ based the model we fitted on heat index levels. Panel (B) shows the odds ratio for hospitalization for changes to the median level of heat index, considering the estimates of PM_2.5_ based on the fitted model on heat index and using the nonlinear interaction model previously estimated.

Additionally, we accounted for the association between the two exposures as described in the Methods, quantifying the hospitalization risk using the estimated coefficients of the nonlinear interaction model and plugging in the PM_2.5_ estimates based on the heat index. Higher levels of PM_2.5_ were associated with higher levels of heat index as shown in Figure 4A, for the median value of heat index the expected value of PM_2.5_ was 11.99 µg/m^3^ (95% CI = 11.97, 12.00 µg/m^3^) based on the fitted regression, while for the extreme heat index the expected value of PM_2.5_ was 16.45 µg/m^3^ (95% CI = 16.42, 16.48 µg/m^3^). The analysis was consistent with earlier findings from the interaction nonlinear model. We estimated an OR of 1.016 (95% CI = 1.001, 1.032) for all-cause hospitalization on extreme heat days compared with the median when simultaneously adjusting for the expected change in PM_2.5_, Figure 4B. Additionally, we carried out a sensitivity analysis, replicating all the analyses just presented, using a 3-day moving average of heat index and PM_2.5_ levels as exposure variables. The results presented a change in trend but were consistent with the main analyses in terms of estimated OR. The sensitivity analysis carried out by running the linear interaction model in two temporal subcohorts (hospitalization during 2000–2007 and 2008–2016) was in line with the main analysis results, showing that the estimated effects are not affected by temporal trends in PM_2.5_. More details on the sensitivity analyses are in the Supplementary Materials Section S4; https://links.lww.com/EE/A386.

## Discussion

In this study, we investigated the impact of heat and PM_2.5_ exposure on all-cause hospitalization risk within a cohort of enrollees previously hospitalized with ADRD across the contiguous US. The impact of the exposures was analyzed both independently and jointly. We found that elevated heat index levels, especially during extreme heat events, were associated with increased hospitalization risk. We found evidence of a nonlinear relationship with PM_2.5_, which indicates a greater increase in hospitalization risk for less exposed enrollees as PM_2.5_ levels rise. We then explored the interaction between heat and PM_2.5_ and lastly accounted for the PM_2.5_-heat index association in assessing their impact on all-cause hospitalizations. We identified a synergistic interaction effect, which multiple biological mechanisms could explain. Heat can increase the body’s uptake of PM_2.5_ by promoting deeper breathing and vasodilation, which enhances pollutant absorption. Both stressors independently trigger oxidative stress and systemic inflammation, and combined exposure might amplify these responses, resulting in greater cardiovascular and respiratory stress. Additionally, heat-induced autonomic dysfunction and impaired thermoregulation exacerbate vulnerability to PM_2.5_-related damage, increasing risks of events like heart attacks and respiratory exacerbations. All steps of the analysis returned consistent results, confirming that these exposures are associated with hospitalizations for a cohort of enrollees affected by ADRD.

The literature consistently demonstrates that, in the general population, short-term heat exposure significantly increases the risk of ADRD-related hospitalization.^17,21–23^ Similarly, studies by Franco et al.^27^ and Zhang et al.^28^ report associations between short-term PM_2.5_ exposure and ADRD-related hospitalizations in the general population. Our study extends this body of work by examining single-exposure associations with all-cause hospitalization in enrollees previously diagnosed with ADRD and analyzing the combined effects of heat and PM exposure on these hospitalizations. While our all-cause hospitalization outcome encompasses those explored in previous studies, our research uniquely focuses on a cohort of enrollees with a previous hospitalization with an ADRD-related code. Despite this difference in focus, our single-exposure findings align with existing literature. For example, Delaney et al.^23^ estimated that the OR of ADRD-related hospitalization on days with heat index at the 99th percentile, compared with days at the 50th percentile, was 1.017 (95% CI = 1.011,1.023), for older adults living in the contiguous US. This result aligns with our estimated OR of 1.017 (95% CI = 1.004, 1.031) for hospitalization on extreme heat days (99th percentile) versus median heat index days. Our single-exposure nonlinear results also indicate a higher risk of all-cause hospitalization linked to increased PM_2.5_ exposure among enrollees previously identified with ADRD, while Franco et al.^27^ estimated a 3.75% (95% CI = 1.91, 5.63) increase in AD-related hospitalizations for a 10 µg/m^3^ increase in PM_2.5_ in Lisbon. Despite the different study designs, these results agree on indicating a harmful effect of exposure to PM_2.5_. Our study is, to the best of our knowledge, the first to investigate the interactions between PM_2.5_ and extreme heat within an ADRD cohort, corroborating prior literature by identifying a detrimental impact.

This study has several limitations. First, our analysis focused on same-day exposure levels, with a 3-day moving average as a sensitivity analysis, yet existing literature indicates that the effects of heat and air pollution can manifest over various lag times.^23,42,51–53^ Even though the sensitivity analysis provided similar results to the main one, future research should investigate the joint effects of heat and air pollution, incorporating multiple lag days. Additionally, we concentrated on heat index and PM_2.5_, although other pollutants like NO_2_ also adversely affect health, particularly in individuals with ADRD, and further research is needed. For example, Dong et al.^54^ identified strong associations between NO_2_ exposure and ADRD-related emergency department visits, and temperature could potentially modify NO_2_ levels.^55^ The chemical structure of PM_2.5_ varies widely due to different sources and has been shown to be related to the impact on health.^56–59^ Additional research is needed to examine how different forms of PM_2.5_ differentially impact risk of hospitalizations for those with ADRD, including under conditions of high heat.

The study population comprises individuals with a documented history of hospitalization with ADRD, enabling assessment of the impact of heat and air pollution exposures on all-cause hospitalizations among this particularly vulnerable group. Due to data limitations—specifically the availability of only Medicare Part A hospitalization records—we identified ADRD cases based on their first ADRD-related hospital admission. This approach may preferentially select individuals with more severe or advanced disease, as it excludes patients with ADRD who have not had a hospitalization, potentially introducing selection bias. Access to broader clinical data, such as outpatient or EHR, would enhance cohort ascertainment and generalizability in future research. This study restricts its analysis to hospitalizations among enrollees who do not reside in skilled nursing facilities, thereby selecting a cohort less likely to be shielded from exposure to air pollution and heat. This approach aims to more accurately characterize the relationship between environmental exposures and hospitalization risk. Future research is warranted to compare outcomes between community-dwelling and skilled nursing facility populations, to determine whether differential exposure or vulnerability exists between these subgroups.

Although our study focused on national-level models to provide an overall assessment, we acknowledge that regional variation in environmental exposures and population characteristics may influence associations. Future research incorporating region-specific analyses could better elucidate potential geographic heterogeneity and improve localized risk estimation in this vulnerable ADRD population. Moreover, by calculating heat index percentiles within climate subtypes, we aimed to partially account for differences in local temperature distributions and population acclimatization. We acknowledge, however, that climate zones remain relatively large and heterogeneous, both in terms of temperature and neurocharacteristics, and that utilizing finer spatial scales and investigating other factors could provide a more nuanced characterization. Lastly, the goal of the paper is to understand the combined effect of these exposures. Although beyond the scope of the current manuscript, future studies should more extensively examine mediation and interaction regarding these exposures, following the approach proposed by VanderWeele.^60^

This study has notable strengths. It utilized a large, nationally representative sample of fee-for-service Medicare beneficiaries spanning 17 years of hospital admissions. The use of a case-crossover design allowed for adjustment of time-invariant factors. Furthermore, the study’s incremental approach in modeling complexity serves as a framework for future research, enhancing understanding of the impact and dependency between two exposures. Although we assessed joint effects through interactions, future work could also include rigorous mediation analysis to further elucidate the relationship between these exposures and hospitalization rates. Such an approach could provide deeper insights into whether the influence of heat on enhancing PM_2.5_ levels plays a mediating role in the observed association with hospital admissions.

In conclusion, this study provides significant insights into the independent and joint effects of heat index and PM_2.5_ exposure on all-cause hospitalizations among enrollees with a prior ADRD hospitalization across the contiguous US. We found that joint elevated levels of heat and PM_2.5_ are associated with increased all-cause hospitalization risk, particularly during extreme heat events. Our findings highlight the importance of considering the interaction between heat and air pollution, as their combined effects may exacerbate health outcomes in this vulnerable population. Despite the study’s limitations, the analysis offers a robust framework for future research. Our work underscores the critical need for targeted interventions and policies that address multiexposure risks in at-risk populations, such as those with ADRD.

## Conflicts of interest statement

The authors declare that they have no conflicts of interest with regard to the content of this report.

## Supplementary Material


