# Supplementary material for: Combined and synergistic effects of heat and fine particulate matter on hospitalization among patients with Alzheimer’s disease and related dementias
Source: Environ Epidemiol. 2025 Nov 21;9(6):e440. doi: 10.1097/EE9.0000000000000440 (PMC12643645; doi:10.1097/EE9.0000000000000440)
Supplement: Supplementary file 1 [file ee9-9-e440-s001.pdf]

Supplementary Material

Combined and Synergistic Effects of Heat and Fine Particulate Matter on Hospitalization Among Patients With Alzheimer's Disease and Related Dementias

Anonymous et al.

Contents

S1 Additional tables **2**

S2 Cohort's inclusion criteria **3**

S3 Additional details on the dependency analysis **4**

S4 Sensitivity analysis **5**

S4.1 3-day average exposures 5

S4.1.1 Single exposure analysis 5

S4.1.2 Joint-exposure analysis 6

S4.2 Separate time windows analysis 8

S5 Additional figures **11**

S1 Additional tables

ICD code	Description
ICD-9	
2900	Senile dementia, uncomplicated
29010	Presenile dementia, uncomplicated
29011	Presenile dementia with delirium
29012	Presenile dementia with delusional features
29013	Presenile dementia with depressive features
29020	Senile dementia with delusional features
29021	Senile dementia with depressive features
2903	Senile dementia with delirium
29040	Vascular dementia, uncomplicated
29041	Vascular dementia, with delirium
29042	Vascular dementia, with delusions
29043	Vascular dementia, with depressed mood
2940	Amnestic disorder in conditions classified elsewhere
29410	Dementia in conditions classified elsewhere without behavioral disturbance
29411	Dementia in conditions classified elsewhere with behavioral disturbance
29420	Dementia, unspecified, without behavioral disturbance
29421	Dementia, unspecified, with behavioral disturbance
2948	Other persistent mental disorders due to conditions classified elsewhere
3310	Alzheimer's disease
33111	Pick's disease
33119	Other frontotemporal dementia
3312	Senile degeneration of brain
3317	Cerebral degeneration in diseases classified elsewhere
797	Senility without mention of psychosis
ICD-10	
F0150	Vascular dementia, unspecified severity, without behavioral disturbance, psychotic disturbance, mood disturbance, and anxiety
F0151	Vascular dementia, unspecified severity, with behavioral disturbance
F0280	Dementia in other diseases classified elsewhere, unspecified severity, without behavioral disturbance, psychotic disturbance, mood disturbance, and anxiety
F0281	Dementia in other diseases classified elsewhere, unspecified severity, with behavioral disturbance
F0390	Unspecified dementia, unspecified severity, without behavioral disturbance, psychotic disturbance, mood disturbance, and anxiety
G300	Alzheimer's disease with early onset
G301	Alzheimer's disease with late onset
G308	Other Alzheimer's disease
G309	Alzheimer's disease, unspecified
G3101	Pick's disease
G3109	Other frontotemporal neurocognitive disorder
G311	Senile degeneration of brain, not elsewhere classified
G312	Degeneration of nervous system due to alcohol
R4181	Age-related cognitive decline

Table S1: ADRD-related ICD-9 and ICD-10 billing codes according to the Moura & Festa refined definition of Moura et al. [1].

S2 Cohort's inclusion criteria

This section provides more details on the inclusion criteria used to define our cohort for this study, showing the change in numerosity at each step. We started by considering all Medicare fee-for-service (Medicare-FFS) enrollees aged 65 and older from January 1, 2000, to December 31, 2016, across the contiguous US.

1. *Qualifying hospitalization* (entering the ADRD cohort): enrollees who had a first hospitalization with at least one ADRD-related diagnosis code in the first ten billing codes, based on the refined definition by Moura et al. [1] (see Table S1). (N = 8.008.391)

2. *Outcome*: enrollees who had a qualifying hospitalization and had another all-cause hospitalization at least 30 days after the discharge day of the qualifying hospitalization. This all-cause hospitalization is defined as "outcome". (N = 3.969.903)

3. Filter on enrollees who were not admitted from nursing facilities for their outcome. (N =3.595.396)

4. Filter on enrollees whose outcome occurred during the warm season, defined as the period from May 1 to September 30. (N = 1.457.109)

5. Filter on enrollees who do not have additional hospitalizations within 30 days from the outcome's discharge date. (N = 721.724)

6. Remove enrollees with missing information on climate or meteorological data. (N = 713.007)

S3 Additional details on the dependency analysis

The final stage of our co-exposure analysis involved examining the association between heat index and $PM_{2.5}$ and including it when assessing their impact on all-cause hospitalizations among ADRD enrollees. This involved two steps: first, predicting $PM_{2.5}$ levels as a function of heat index and second evaluating odds for hospitalization for a change in heat index as well as the predicted change in $PM_{2.5}$. The two models employed for this analysis are structured as follows:

$$PM_{2.5} = \beta_{BS} \cdot BS(HI,\ 4\ df) \tag{S1}$$

$$\begin{aligned} case = {} & \beta_{BS} \cdot BS(HI,\ 3\ df) + \beta_{BS} \cdot BS(HI,\ 3\ df) \\ & + \beta_{CB} \cdot CB(HI,\ PM_{2.5}) \end{aligned} \tag{S2}$$

Here, the coefficients β_{BS} in (S1) reflect the relationship between the heat index and $PM_{2.5}$, the coefficients β_{BS} in (S2) reflect the independent associations between the exposures and the hospitalizations, while β_{CB} denote the joint associations between both exposures and hospitalization risk. This dual-model approach enables a comprehensive evaluation of the interplay between heat and air pollution, and their combined impact on health outcomes within the ADRD cohort.

We evaluated hospitalization rate differences between median and extreme heat days (99th percentile), accounting for the heat-$PM_{2.5}$ relationship, i.e., This estimand is described as follows,

$$\begin{aligned} & E[Y|\text{heat} = .99, PM_{2.5} = E\{PM_{2.5}|\text{heat} = .99\}] - \\ & \qquad E[Y|\text{heat} = .5, PM_{2.5} = E\{PM_{2.5}|\text{heat} = .5\}]. \end{aligned}$$

For the analysis incorporating expected $PM_{2.5}$ changes based on heat index levels, we used a Monte Carlo procedure with 5,000 iterations to account for uncertainty from two models. In each iteration, random realizations of the coefficients were generated based on their expected values and variances to first estimate $PM_{2.5}$, and then assess ORs.

S4 Sensitivity analysis

To assess sensitivity, in Section S4.1, we repeated analyses using the average exposure from lag 0 to 2 days for heat index percentile and $PM_{2.5}$, rather than same-day levels. Results are presented as OR for all-cause hospital admissions, with reference levels of 5 $\mu g/m^3$ for average $PM_{2.5}$ and 50% percentile for the average heat index. In Section S4.2, we split the data based on two time windows and compared both the distribution of the exposures and the results of the joint-exposure linear interaction model in order to check the sensitivity of the model with respect to the decreasing temporal trend of $PM_{2.5}$.

S4.1 3-day average exposures

S4.1.1 Single exposure analysis

Assessing the linear relationship, we estimated an OR of 1.008 (95% CI: 1.001, 1.016) for hospitalization on extreme heat days (99th percentile, i.e. a 49% percentile increase) versus median temperature days and an OR of 1.001 (95% CI: 0.999, 1.004) for 5 $\mu g/m^3$ increase in $PM_{2.5}$. Non-linear analysis showed a steeper trend for higher values of heat index averages, with an OR of 1.030 (95% CI: 1.012, 1.047) for extreme heat days compared to the median. $PM_{2.5}$ did not present an association with hospitalizations, we estimated an OR of 1.001 (95% CI: 0.994, 1.008) for 10 $\mu g/m^3$ versus 5 $\mu g/m^3$ and of 1.001 (95% CI: 0.997, 1.005) for 15 $\mu g/m^3$ versus 10 $\mu g/m^3$. Results for both linear and non-linear associations are presented in Table S2.

	**Model**	**$PM_{2.5}$**	**Heat Index**
**Single Exposure**	Linear	1.001 (0.999, 1.004)	1.008 (1.001, 1.016)
	Non-Linear	1.001 (0.994, 1.008)	1.030 (1.012, 1.047)
**Joint-Exposure**	Linear	1.000 (0.997, 1.003)	1.009 (1.000, 1.017)
	Non-Linear	0.998 (0.991, 1.006)	1.030 (1.012, 1.047)

Table S2: **Odds ratio for all-cause hospitalization.** The table presents the odds ratio for hospitalization in moving from the median level of heat index to extreme heat days (99th percentile) and from a 5 $\mu g/m^3$ level of $PM_{2.5}$ to 10 $\mu g/m^3$. The odds ratios are estimated both using single exposure models and joint-exposure model without interaction.

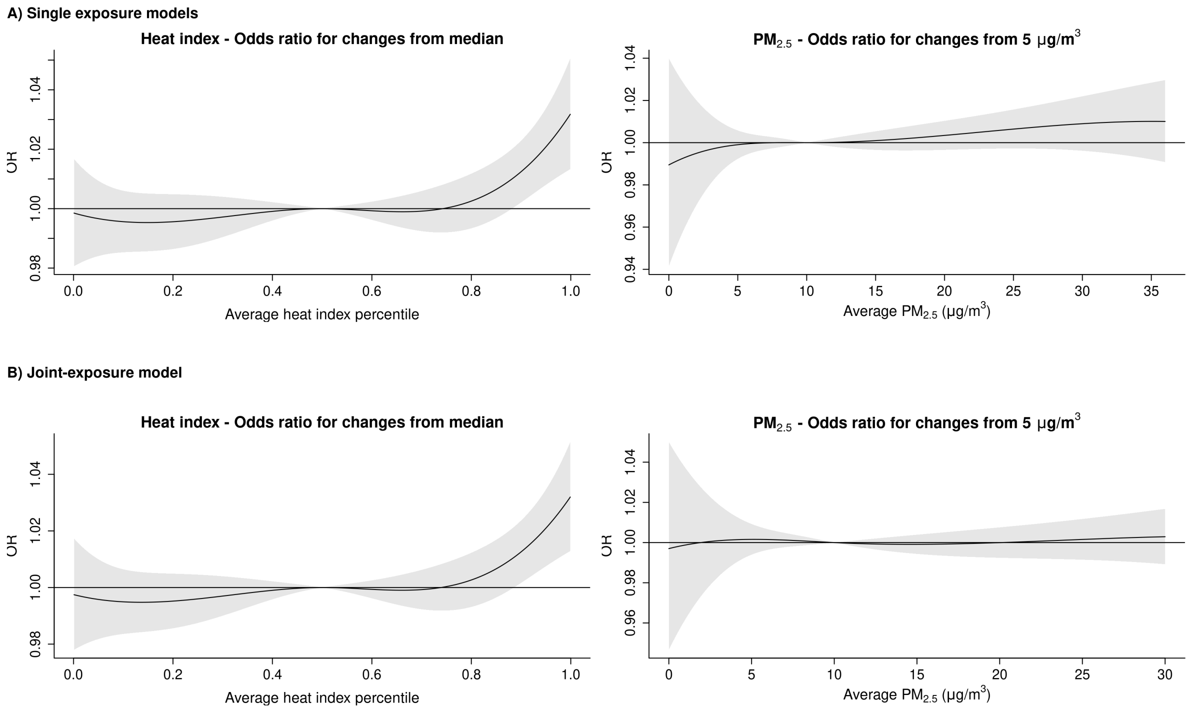

Figure S1: **Odds ratio for all-cause hospitalization.** The plots present the estimated non-linear odds ratio for hospitalization in moving from the median level of heat index and from a 5 $\mu g/m^3$ of $PM_{2.5}$. Panel A shows the odds ratio obtained from the single exposure models, panel B shows the odds ratio obtained from the joint-exposure model.

S4.1.2 Joint-exposure analysis

In the linear joint-exposure model without interaction, the OR for hospitalization was 1.009 (95% CI: 1.000, 1.017) for extreme heat days against the median and 1.000 (95% CI: 0.997, 1.003) for a 5 $\mu g/m^3$ increase in $PM_{2.5}$. The non-linear analysis confirmed the lack of association between $PM_{2.5}$ and hospitalizations, and a stronger effect of the heat index at extreme values, as shown in Figure S1A. The OR of hospitalization was 1.030 (95% CI: 1.012, 1.048) for extreme heat days compared to median temperatures. For $PM_{2.5}$, the OR was 0.998 (95% CI: 0.991, 1.006) at 10 $\mu g/m^3$ versus 5 $\mu g/m^3$, and 0.999 (95% CI: 0.994, 1.004) at 15 $\mu g/m^3$ versus 10 $\mu g/m^3$. The results are reported in Table S2 and shown in Figure S1B.

Considering the lag 0 to lag 2 averages diminished the interaction effect we observed in the main analysis, especially for high levels of heat index where we observed an association with hospitalizations that was constant across different $PM_{2.5}$ levels.

Specifically, through the linear analysis, we estimated a marginal OR of 1.005 (95% CI: 0.992, 1.018) on extreme heat days compared to the median and 0.998 (95% CI: 0.991, 1.004) for $PM_{2.5}$ at 10 $\mu g/m^3$ versus 5 $\mu g/m^3$.

Through the non-linear analysis, we estimated an OR of 0.995 (95% CI: 0.986, 1.005) for $PM_{2.5}$ at 10 $\mu g/m^3$ versus 5 $\mu g/m^3$ at the 50th heat index percentile that rose to 1.001 (95% CI: 0.976, 1.026) at the 99th heat index percentile. The OR for changing heat index from median to 99th percentile was 1.025 (95% CI: 0.986, 1.065) at 5 $\mu g/m^3$ $PM_{2.5}$, increasing to 1.031 (95% CI: 1.011, 1.052) at 10 $\mu g/m^3$. The OR was 1.026 (95% CI: 1.004, 1.048) jointly moving from median heat index and 5 $\mu g/m^3$ $PM_{2.5}$ to extreme heat and 10 $\mu g/m^3$ $PM_{2.5}$. Figure S2 illustrates the OR associated with variations in exposure as compared to the reference levels, defined as 5 $\mu g/m^3$ for average $PM_{2.5}$ and the median average heat index, Figure S6 presents the 95% confidence intervals.

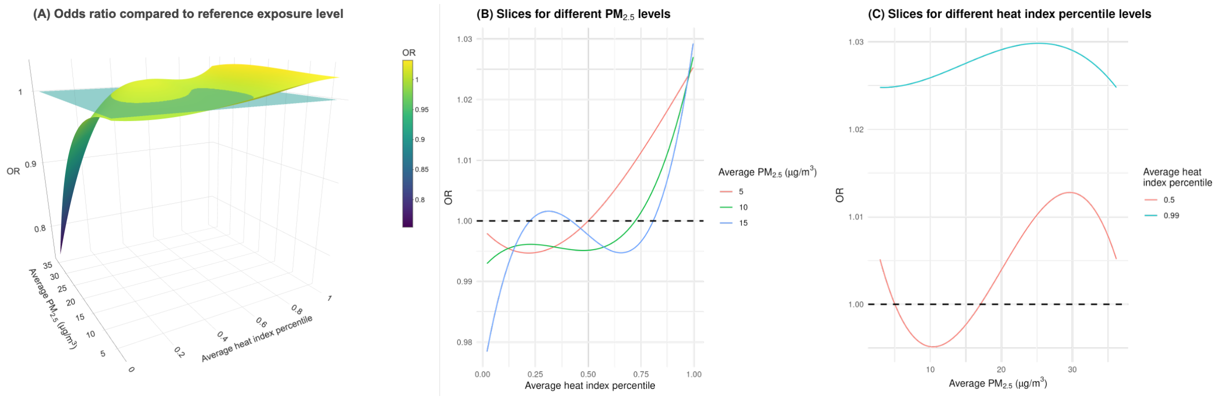

Figure S2: **Joint-exposure non-linear interaction model**. The plots present the estimated non-linear OR for all-cause hospitalizations compared to the median level of heat index and 5 $\mu g/m^3$ of $PM_{2.5}$, accounting for their interaction. Panel (A) shows the OR for the different values of heat index and $PM_{2.5}$ with respect to 5 $\mu g/m^3$ of $PM_{2.5}$ and median value of heat index, the blue plane shows where the OR is equal to 1. Panels (B) and (B) show slices of the first plot. Specifically, panel (B) shows the OR for exposure levels compared to 5 $\mu g/m^3$ of $PM_{2.5}$ and the median level of heat index, for heat index values fixed at the median, and 99% percentile. Panel (C) shows the OR for exposure levels compared to 5 $\mu g/m^3$ of $PM_{2.5}$ and the median level of heat index, for fixed values of $PM_{2.5}$ equal to 5, 10, and 15 $\mu g/m^3$.

To conclude this sensitivity analysis we repeated the analysis accounting for the dependency between the exposures. Higher heat levels predicted higher $PM_{2.5}$ concentrations (Figure S3A), for the median value of heat index the expected value of $PM_{2.5}$ was 12.00 $\mu g/m^3$ (95% CI: 11.99, 12.01 $\mu g/m^3$), while for the extreme heat index the expected value of $PM_{2.5}$ was 15.31 $\mu g/m^3$ (95% CI: 15.27, 15.34 $\mu g/m^3$). Accounting for their interaction, the OR for hospitalization on extreme heat

days compared to median days was 1.033 (95% CI: 1.012, 1.055), confirming a higher hospitalization risk with increased heat exposure.

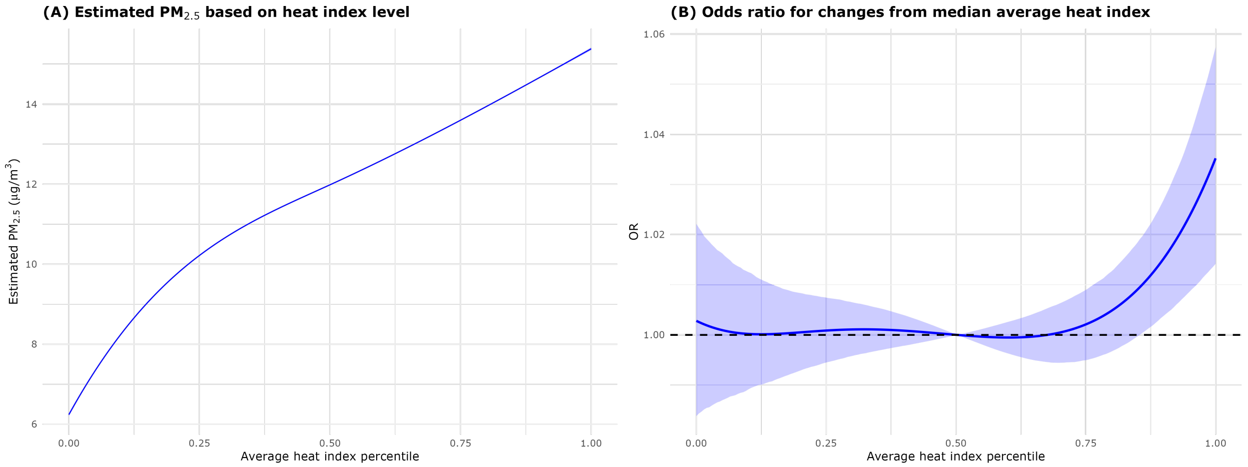

Figure S3: **Association between $PM_{2.5}$ and heat index and association with all-cause hospitalizations.** Panel (A) shows the association between $PM_{2.5}$ and heat index presenting the estimated values of $PM_{2.5}$ based the model we fitted on heat index levels. Panel (B) shows the odds ratio for hospitalization for changes to the median level of heat index, considering the estimates of $PM_{2.5}$ based on the fitted model on heat index.

This sensitivity analysis estimated ORs that generally align with those from the main analysis but modifies some observed trends. In the main analysis, the association between the heat index and hospitalizations exhibited a linear trend, which became steeper at higher values in the sensitivity analysis. Conversely, while the main analysis showed $PM_{2.5}$ having a pronounced effect at low concentrations, plateauing for exposures above 10 $\mu g/m^3$, the sensitivity analysis did not present this initial stronger effect.

S4.2 Separate time windows analysis

To assess whether temporal changes in $PM_{2.5}$ concentrations influenced the study results, we conducted a sensitivity analysis examining both the exposure distribution across years and the robustness of the linear interaction model in subcohorts split by calendar time.

Table S3 presents the annual summary statistics of $PM_{2.5}$ exposure across the study period (2000–2016). Reported metrics include the minimum, maximum, mean, median, standard deviation, and the 5th and 95th percentiles. A general declining trend in the mean and median $PM_{2.5}$

levels is observed, while the overall range of exposures remains stable across years. Specifically, the minimum exposure values were constant over the study period, and though maximum values fluctuated, no clear increasing or decreasing trend was present. This indicates that the exposure–response relationship remains adequately supported, as the same range of exposure is consistently represented across years.

Year	Min	5th perc	Mean	Median	95th perc	Max	SD
2000	0.35	4.52	13.67	12.29	27.69	119.87	7.29
2001	0.00	4.20	13.90	11.76	30.78	71.10	8.59
2002	0.60	4.19	14.04	11.82	31.63	134.35	9.09
2003	0.38	4.61	13.59	11.86	28.61	99.80	7.99
2004	0.32	4.33	13.20	11.48	27.20	71.37	7.54
2005	0.37	4.21	13.91	11.99	30.19	84.13	8.29
2006	0.26	4.26	12.96	11.51	26.37	72.32	7.10
2007	0.17	4.13	13.67	12.00	28.81	75.11	7.86
2008	0.29	3.96	11.54	10.40	22.52	168.16	6.32
2009	0.45	3.72	9.98	9.16	18.89	85.93	4.74
2010	0.14	3.52	10.22	9.40	19.89	52.27	5.20
2011	0.02	3.47	10.61	9.49	21.53	70.76	5.71
2012	0.00	3.62	9.26	8.63	16.79	110.01	4.20
2013	0.21	3.10	8.75	8.11	16.55	160.30	4.42
2014	0.00	3.38	8.81	8.23	16.37	100.65	4.08
2015	0.12	3.05	8.77	7.96	16.91	185.18	4.83
2016	0.00	3.01	7.60	7.11	13.75	79.97	3.43

Table S3: **Summary statistics of $PM_{2.5}$ exposure across the years of the study.** The table reports the minimum, maximum, mean, median, standard deviation, and 5th and 95th percentiles of $PM_{2.5}$ exposure for every year of the study.

To evaluate the potential impact of observed temporal trends, we divided the study cohort into two time windows: 2000–2007 (early period) and 2008–2016 (later period). We compared both the exposure distribution and the results of the joint-exposure linear interaction model across these subcohorts. Figure S4 displays the joint distributions of $PM_{2.5}$ and heat index for the two time periods. $PM_{2.5}$ concentrations were generally lower in the later years, but the joint distributions showed similar overall patterns between the two windows. The main difference was a lower frequency of extreme $PM_{2.5}$ values in the more recent period.

Table S4 reports the estimated marginal ORs for all-cause hospitalization, comparing a 5-unit increase in $PM_{2.5}$ at the 49th percentile of the heat index. The results were highly consistent across

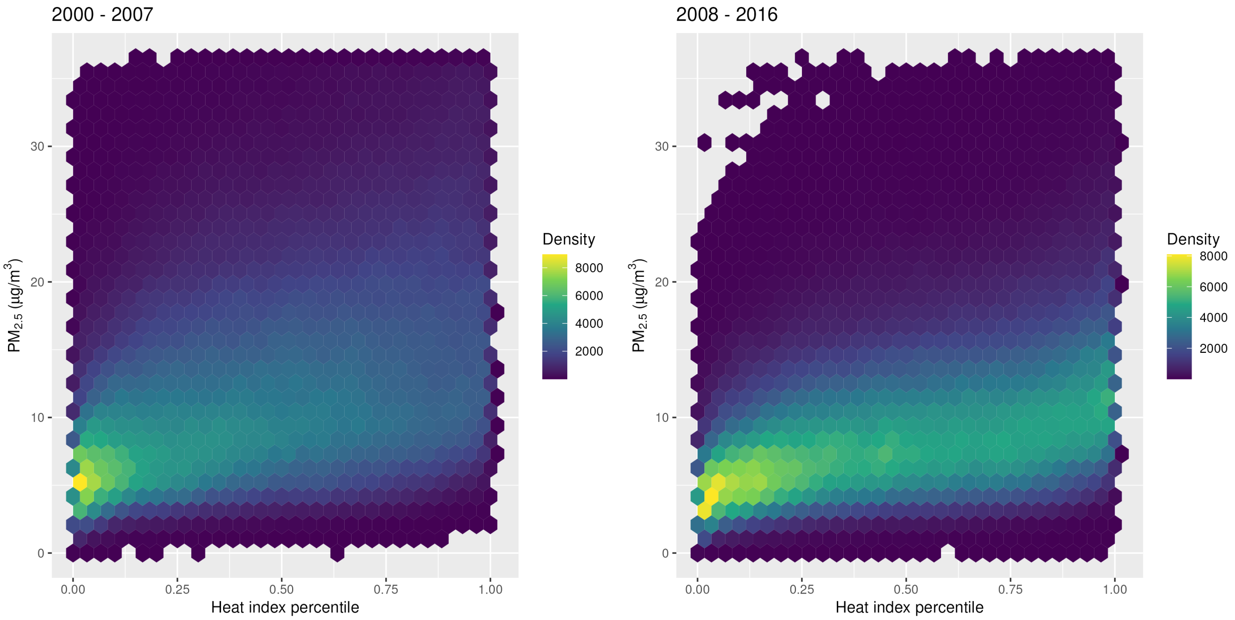

Figure S4: **Joint distribution of $PM_{2.5}$ and heat index, splitting the data based on two time windows.** The plot shows the joint density of heat index and $PM_{2.5}$ considering two time windows: 2000–2007 and 2008–2016. Both plots present values of $PM_{2.5}$ up to the 99th percentile of its complete distribution, i.e., 36.18 $\mu g/m^3$.

subcohorts and aligned closely with the main analysis, indicating that the declining long-term trend in $PM_{2.5}$ did not affect the validity of the estimated associations.

	2000–2007	2008–2016
$PM_{2.5}$	1.0035 (0.9967, 1.0103)	1.0090 (0.9982, 1.0198)
Heat index	1.0230 (1.0069, 1.0393)	1.0244 (1.0072, 1.0420)
Interaction term	0.9973 (0.9922, 1.0024)	0.9933 (0.9858, 1.0008)

Table S4: **Linear interaction model, splitting the data based on two time windows.** The table reports the estimated marginal odds ratio for all-cause hospitalization for a 49th percentile increase in heat index and a 5-unit increase in $PM_{2.5}$ using the linear joint-exposure interaction model, dividing the cohort into two time windows: 2000–2007 and 2008–2016.

S5 Additional figures

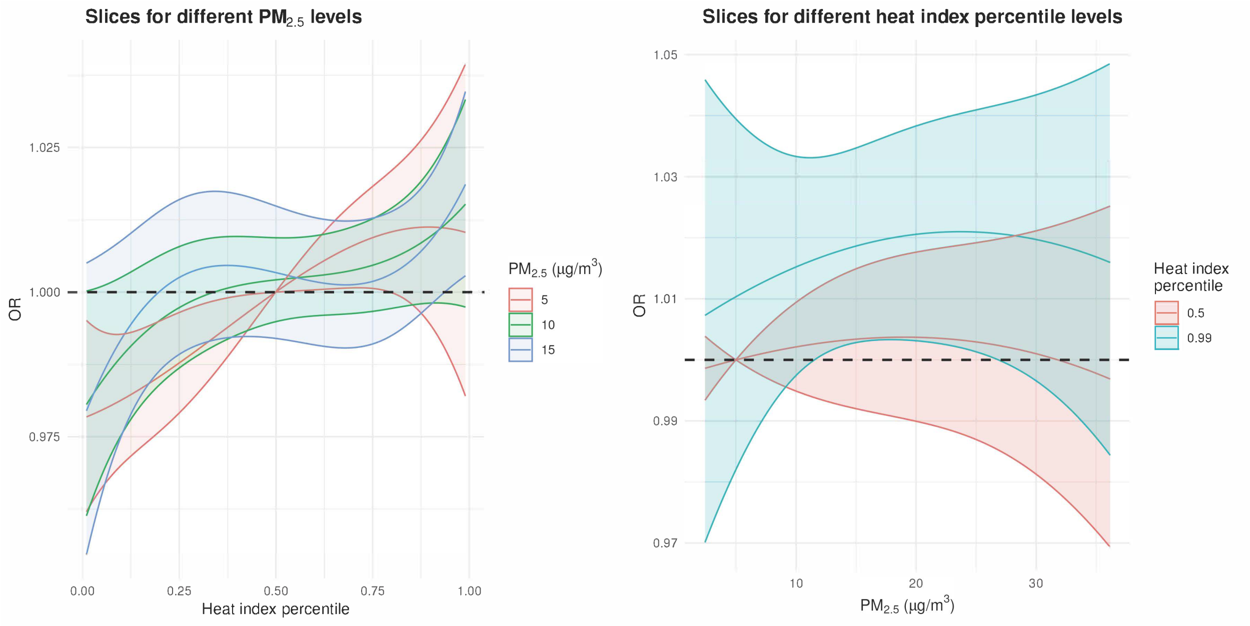

Figure S5: **Joint-exposure non-linear interaction model**. The plots present the estimated non-linear OR for hospitalization in moving from the median level of heat index and from a 5 $\mu g/m^3$ of $PM_{2.5}$, accounting for their interaction. It reports the same plot in Figure 3 in the main manuscript with 95% confidence intervals.

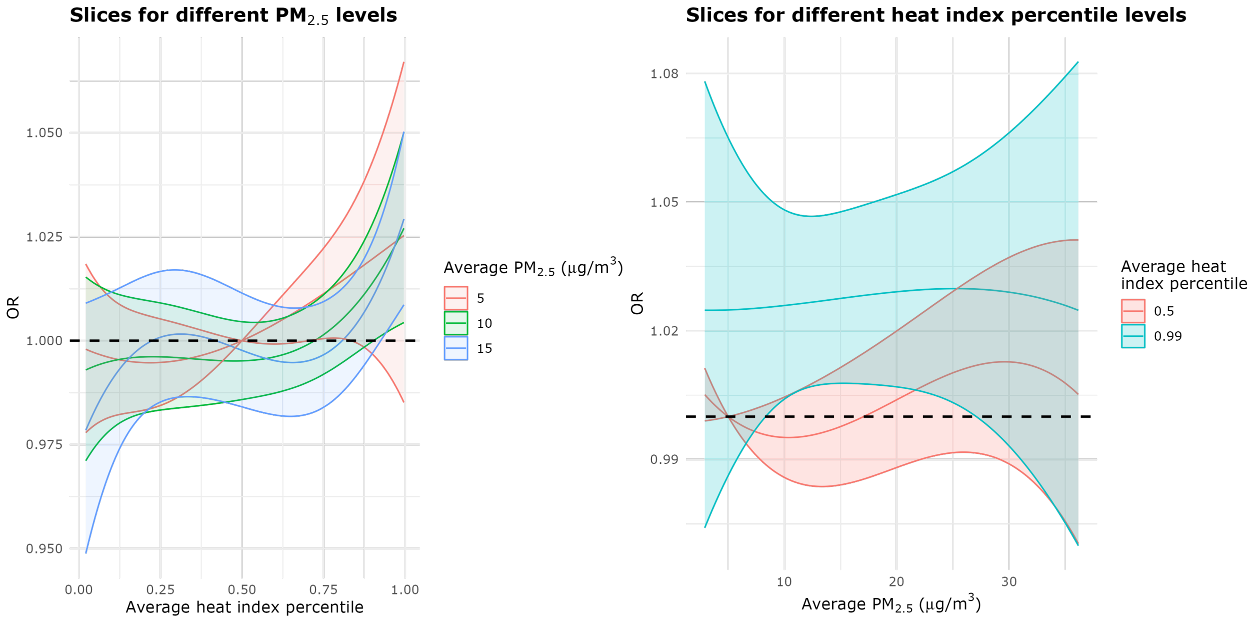

Figure S6: **Joint-exposure non-linear interaction model in sensitivity analysis**. The plots present the estimated non-linear OR for hospitalization in moving from the median level of heat index and from a 5 $\mu g/m^3$ of $PM_{2.5}$, accounting for their interaction. It reports the same plot in Figure S2 with 95% confidence intervals.

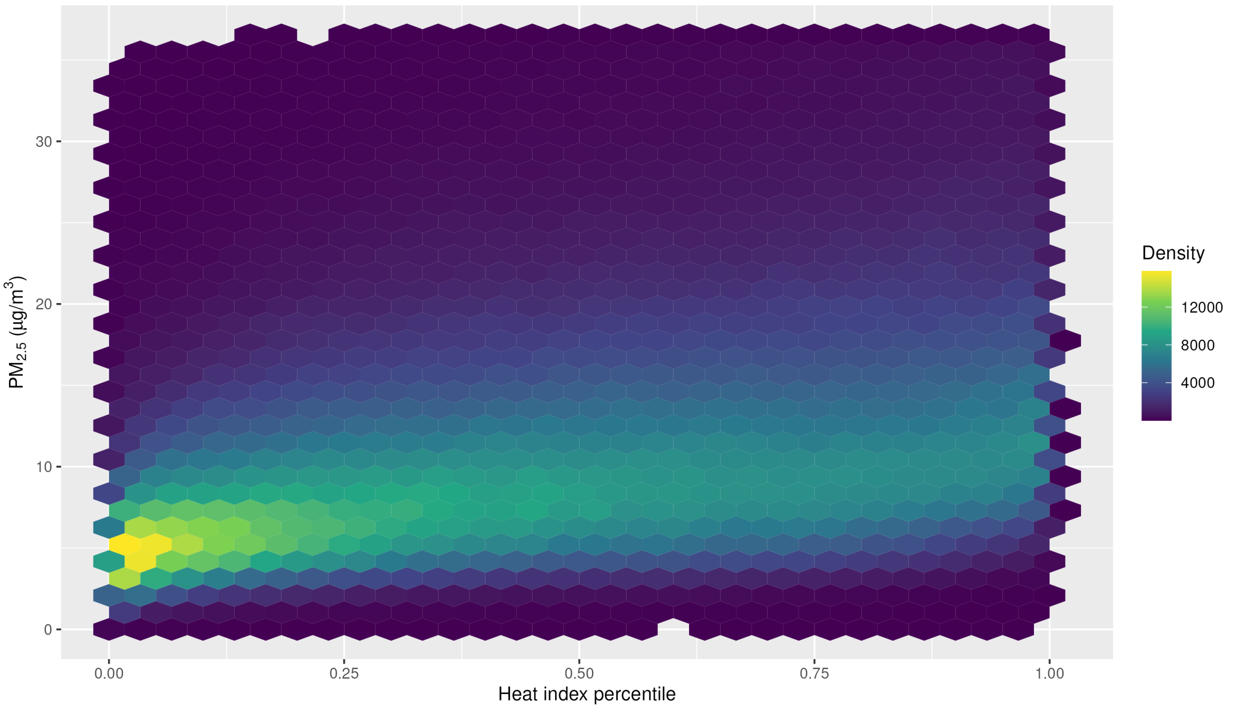

Figure S7: **Joint distribution of $PM_{2.5}$ and heat index.** The plot shows the joint density of heat index and $PM_{2.5}$. The plot presents values of $PM_{2.5}$ up to the 99th percentile of its complete distribution, i.e., 36.18 $\mu g/m^3$.

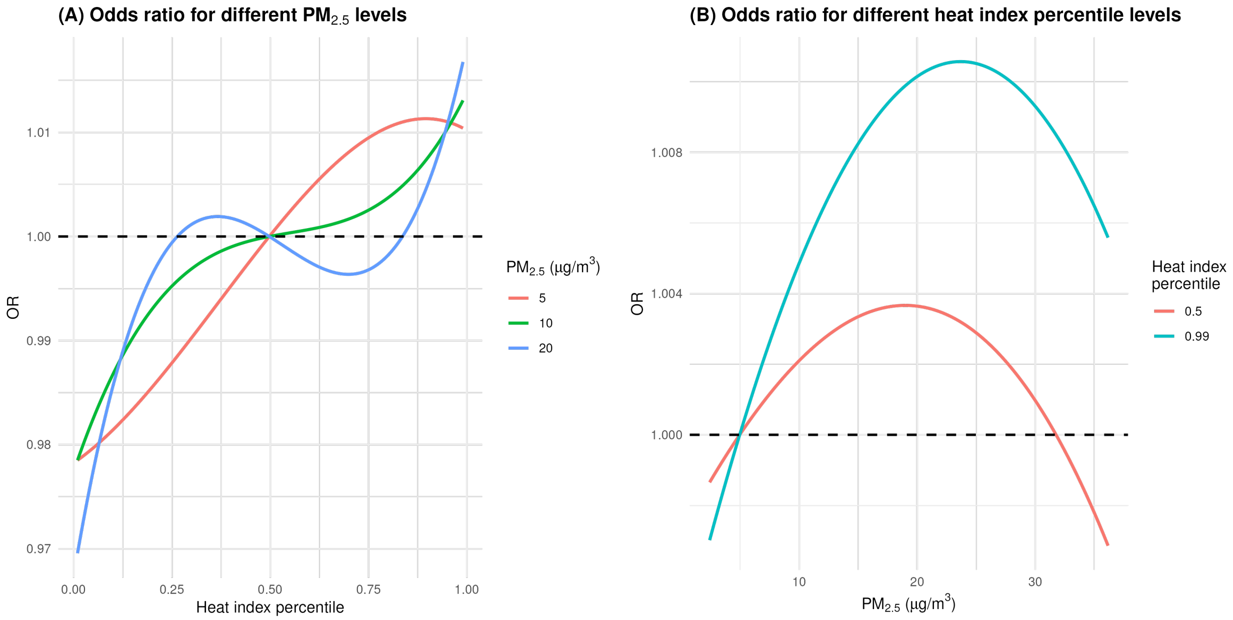

Figure S8: **Joint-exposure non-linear interaction model for different reference levels.** The plots present the estimated non-linear OR for all-cause hospitalizations, accounting for their interaction, considering different reference values. Panel (B) shows the OR for different exposure levels of heat index compared to the reference level given by the median level of heat index and (red curve) 5 $\mu g/m^3$ of $PM_{2.5}$, (green curve) 10 $\mu g/m^3$ of $PM_{2.5}$, and blue curve) 15 $\mu g/m^3$ of $PM_{2.5}$. Panel (C) shows the OR for exposure levels of $PM_{2.5}$ compared to the reference level given by 5 $\mu g/m^3$ of $PM_{2.5}$ and (red curve) the median level of heat index, and (blue curve) 99th percentile of heat index.

References

[1] L. M. V. R. Moura, N. Festa, M. Price, et al. Identifying medicare beneficiaries with dementia. *Journal of the American Geriatrics Society*, 69(8):2240–2251, Apr. 2021. ISSN 1532-5415. doi: 10.1111/jgs.17183. URL http://dx.doi.org/10.1111/jgs.17183.
